# A retrospective cohort study of factors associated with severity of falls in hospital patients

**DOI:** 10.1038/s41598-022-16403-z

**Published:** 2022-07-18

**Authors:** Manonita Ghosh, Beverly O’Connell, Ebenezer Afrifa-Yamoah, Sue Kitchen, Linda Coventry

**Affiliations:** 1 School of Nursing and Midwifery, Edith Cowan University, 270 Joondalup Drive, Joondalup, WA 6027 Australia; 2 Centre for Nursing Research, Sir Charles Gairdner Osborne Park Health Care Group, North Metropolitan Health Service, Level 1, Harry Perkin’s Building, Sir Charles Gairdner Hospital, 6 Verdun Street, Nedlands, WA 6009 Australia; 3 Centre for Research in Aged Care, Edith Cowan University, 270 Joondalup Drive, Joondalup, WA 6027 Australia; 4 School of Science, Edith Cowan University, 270 Joondalup Drive, Joondalup, WA 6027 Australia; 5 CNC Clinical Lead Falls Management and Chair Falls MD Committee, Sir Charles Gairdner Hospital, Perth, WA 6009 Australia

**Keywords:** Health services, Epidemiology

## Abstract

Severity of falls in hospital patients are threat to patient safety which can result in a financial burden on the patient’s family and health care services. Both patient specific and environmental and organisational factors are associated with severity of falls in hospital. It is important to continuously analyse the factors associated with severity of fall which can inform the implementation of any fall preventive strategies. This study aims to identify factors associated with the severity of falls in hospitalised adult patients in Western Australia. This study involved a retrospective cohort analysis of inpatient falls records extracted from the hospital’s Clinical Incident Database from May 2014 to April 2019. Severity of falls were classified as three Severity Assessment Code (SAC): SAC 1 was “high” causing serious harm or death; SAC 2 was “medium” causing moderate or minor harm; and SAC 3 was “low” indicating no harm. Univariable and multivariable generalised ordinal logistic regression models were used to quantify the magnitude of effects of the potential risk factors on severity of falls at 5% level of significance and reported the crude odds and adjusted odds ratio of falling at a higher severity level. There were 3705 complete reported cases of falls with the average age of the patients was 68.5 ± 17.0 years, with 40.2% identified as female. The risk of falling at a higher level of severity increased by patient age over 50 years. Females were 15.1% more likely to fall at higher severity level compared to females. Fall incidents occurred during toileting and showering activities and incidents in a communal area were 14.5% and 26% more likely to occur at a higher severity respectively. Similarly, depression (167%), influence of alcohol or illicit drugs (more than 300%), use of medications (86%) and fragile skin (75%) significantly increased the odds of falling at higher level of severity. Identification of underlying risk factors associated with fall severity provides information which can guide nurses and clinicians to design and implement effective interventional strategies that mitigate the risk of serious fall injuries. The results suggest that fall prevention strategies should target patients with these risk factors to avoid severity of falls.

## Introduction

Severity of falls in hospitalised patients are a serious concern for patient care. The incidence of inpatient falls ranges from 1.7 to 16.9 per 1000 patient-days^1–4^. Among inpatient falls, the incidence rates for severity of falls and fall-related injury range from 6.8 to 72.1% for mild and 0.7–30% for severe injuries^5^. These severe fall-related injury can include fracture, subdural hematomas, excessive bleeding, cranial trauma, loss of independence or even death^5–7^. Hence, severity of falls are threat to patient safety and quality of life, and responsible for prolonged hospital stay, economic burden on patient family and health care costs^3,8^. Patients with fall-related injury in the US were reported to have hospital charges more than $4200 higher than patients who did not fall^6,9^. Considering the adverse impact, strategies to prevent fall-related injury have been the growing research focus. Yet, fall-related injury remains steady adverse events in acute hospitals^10–12^. Research showed there were 6–26% of the total inpatient falls resulted in serious injuries in the US hospitals^13,14^. It is therefore crucial to examine the nature of fall severity and identify risk factors of severity of falls to decrease serious fall-related injury in acute hospitals.

In Australia, falls were reported causing 37% of all injury deaths, and more than 34,000 hospitalisations (3.2 per 1000 hospitalisations) reported a fall over ten-year trends^15^. The total hospital cost of inpatient falls in 12 acute medical and surgical wards of six Australian hospitals was $9.8 million, with $6.4 million attributed to non-injurious falls and $3.4 million to injurious falls^16^. Each fall was associated with increased additional hospital stay (more than eight days) and incurred over $6669 additional hospital costs^16^. In WA, there were 336 fall-related deaths reported during a 12-month time in 2016 equating to 11.5 death per 100,000 population, and 26,338 fall-related hospitalisations, an age-standardised rate of 960.7 per 100,000 population in 2017^17^. Australia has a national standard on monitoring and reporting patient incident system to support falls prevention in hospitals^18^.

Recognising the risk factors for severity of falls is critical prior to the establishment of preventive interventions. Studies have reported that inpatient falls are multifaceted involving intrinsic factors and extrinsic factors^3,19^. Intrinsic factors include patient age, gender, medical condition, mobility impairment, whereas extrinsic factors include organisational structures and environmental factors^5,20^. Ageing is a well-known risk factor for falls and fall-related injury^13,21^. However, evidence suggests that severity of falls and fall-related serious injury are much more complicated and therefore, examining multilevel factors associated with severity of falls are recommended^22^. Particular attention is therefore needed for the elderly patients to identify the risk factors for fall severity and develop policy and procedures to avoid serious injuries. While there have been several studies on falls prevalence and falls risk factors, little research is published with specific focus on the variables associated with the severity of the fall in adults. This is the first study to examine multivariable factors associated with severity of falls in adult patients in an acute metropolitan tertiary hospital in Western Australia (WA). Identifying the complex relationship of the underlying risk factors and establishing a profile may contribute to the design of effective prevention, improvement and implementing strategies to eliminate or reduce severity of fall and thereby serious fall injurious in the targeted population.

## Methods

### Data source and study design

Data was collected from one of Australia’s leading teaching hospitals in WA situated 4 km from Perth city centre, handling over 76,000 admissions annually with more than 600 beds and employs about 5500 staff treating over 420,000 adult patients each year. The hospital provides a comprehensive range of clinical services including trauma, emergency and critical care, orthopaedics, general medicine, general surgery and cardiac care.

This study involved a retrospective cohort analysis of inpatient falls recorded in the Clinical Incident Management System (CIMS)—an electronic online system implemented by the Department of Health WA (DOHWA) to capture and manage clinical incidents occurred within the WA health system from May 2014 through April 2019. Reporting falls is mandatory through the CIMS database for monitoring and improving patient safety and evaluating the impact of interventions^23^. An inpatient fall was defined as a sudden, unexpected incident in which a patient involuntarily descends from standing, sitting or other horizontal position to the ground or other surface^24^. Inpatient falls were routinely registered into the CIMS by nurses and other hospital employees discovering the fall. All patients were screened and assessed for their risk of falling on admission, and after a fall. Patient information data including demographic, admission, discharge information and clinical characteristics was also reported in the system.

### Variables

Severity of falls were classified as three Severity Assessment Code (SAC) in the CIMS as SAC 1, SAC 2 and SAC 3^23^. A SAC 1 was considered as “high” incident which caused serious harm or death. Patients with SAC 1 had a major surgery and increased the length of hospital stay more than seven days. A SAC 2 was “medium” which had or could have caused moderate or minor harm. Patients with SAC 2 might have a minor fracture or require a minor treatment and increased the length of hospital stay less than 7 days. A SAC 3 was “low” indicating no harm. Patients with SAC 3 had no injuries and the fall did not increase the length of hospital stay.

Intrinsic factors included patient age, gender, behavioural factors including dementia, depression and neurological condition, as well as mobility impairment such as poor balance, and severe foot problems. Extrinsic factors included environmental factors: activity at time of the fall, history of falls, and medication effects; and organisation factors: place of incident, height of fall and intervention in place at the time of the fall. Age was categorised into five groups < 50, 50–64, 65–74, 75–84 and > 84 years. Activity at time of the fall was categorised as attempting to sit, stand, bend; getting in or out of bed; toiling or showering; walking or running; and unknown. Place of incident was reported as bathroom, bed, allied health treatment area, and communal area such as dining room, waiting room, corridor, ground and carpark. Height of fall was divided into low fall (< 0.5 m e.g., an ultra-low bed), medium fall (0.5–1.0 m, e.g., chair or stool), high fall (> 1.0 m, e.g., a high bed) and unknown.

### Data analysis

In the CIMS each patient name was replaced with a Unique-Record-Number (URN) to de-identify them. De-identified records of patients ≥ 18 years who had falls during the study period were extracted from the database. Patients with no URN or were entered after April 2019 were excluded from analysis. Descriptive statistics were calculated for all baseline variables in terms of frequencies and percentages stratified by SAC. We fitted univariable and multivariable generalised ordinal logistic regression models to quantify the magnitude of effects of the variables on SAC and reported the crude odds ratio (OR) and adjusted odds ratio (AOR). In both univariable and multivariable models, the number of falls was used as an offset to correct for any bias in predicting severity of fall. Number of falls as an offset is addressing recurrence effect or time effect, that is, patients fall history may have some form of “maturity effect’ on the outcome of severity that was measured for the most recent fall incident. Missing data for each variable was identified and removed from the analysis. Removing missing data was safe to do so as all deleted rows belonged to SAC3 which was the dominant group. Two-sided p-values < 0.05 of a 95% confidence interval (CI) was considered significant, and all analyses were performed in R v 4.1.1^25^ using the ‘oglmx’ package^26^.

### Ethics approval

This study obtained approval from WA Health for quality improvement (GEKO-33027). This study involved routinely collected only de-identified data by the hospital administrative and was deemed negligible risk. As per the Australian National Health and Medical Research Council’s (NHMRC)^27^ ‘National Statement on Ethical Conduct in Research Involving Humans’ guidelines and regulations (Section 5.1.22) outlining “institutions may choose to exempt from ethical review research that is (a) is negligible risk; and (b) involves the use of existing collections of data or records that contain only non-identifiable data about human beings, the study was exempt from review by the hospital Human Research Ethics Committee. This study was also considered by the Edith Cowan University Human Research Ethics Committee (2019-00653-COVENTRY) as of negligible risk and exempt from ethics review. Consequently, participant’s informed consent was not applicable to this study. Data analysis was carried out in accordance with NHMRC guidelines^27^.

## Results

There were 3705 complete reported cases of falls included in this analysis, of which, 3545 patients suffered low level severity (SAC 3), 142 suffered medium level severity (SAC 2) and 18 suffered high level severity (SAC 1) of falls (Table 1). The average age of the patients was 68.5 ± 17.0 years, with 40.2% identified female. The likelihood ratio and chi-square tests revealed that gender, activity at time of the fall and height of fall were associated with the severity of the fall.

**Table 1:** Comparison of demographic and environmental characteristics and the severity level of fall.

Variables	N = 3705 *n (%)*	Level of severity SAC 3 SAC 2 SAC 1 (n = 3545) (n = 142) (n = 18)			p-value
**Age**					0.216^a^
< 50	508 (13.7)	482 (13.7)	25 (17.6)	1 (5.6)	
50–64	844 (22.8)	808 (22.7)	30 (21.1)	6 (33.3)	
65–74	801 (21.6)	722 (21.9)	25 (17.6)	4 (22.2)	
75–84	868 (23.4)	835 (23.5)	32 (22.5)	1 (5.6)	
> 84	684 (18.5)	648 (18.2)	30 (21.1)	6 (33.3)	
**Gender**					0.029^b^*
Male	2216 (59.8)	2133 (60.1)	77 (54.2)	6 (33.3)	
Female	1489 (40.2)	1412 (39.9)	65 (45.8)	12 (66.7)	
**Activity at the time of fall**					0.042^b^*
Attempting to sit/stand/bending and reaching over	1350 (36.4)	1311 (35.0)	36 (25.4)	3 (16.7)	
Getting in/out of bed	685 (18.5)	652 (17.2)	31 (21.8)	2 (11.1)	
Toileting and showering	869 (23.5)	824 (21.8)	41 (28.9)	4 (22.2)	
Walking and running	676 (18.2)	648 (17.6)	21 (14.8)	7 (38.9)	
Unknown	98 (2.6)	83 (8.4)	13 (9.2)	2 (11.1)	
**Place of incident**					0.380^b^
Bathroom	909 (24.5)	871 (24.5)	33 (25.4)	5 (31.3)	
Bed	1301 (35.1)	1257 (35.3)	41 (31.5)	3 (18.8)	
Allied health treatment area	1356 (36.6)	1301 (36.6)	49 (37.7)	6 (37.5)	
Communal area	139 (3.8)	130 (3.7)	7 (5.4)	2 (12.5)	
**Height of fall**					< 0.001^b^*
Low fall (< 0.5 m)	1460 (39.4)	1436 (37.6)	41 (28.9)	2 (11.1)	
Medium fall (0.5–1.0 m)	1761 (47.5)	1716 (44.9)	67 (48.5)	12 (66.7)	
High fall (> 1.0 m)	31 (0.8)	25 (0.7)	6 (7.2)	0 (0)	
Unknown	453 (12.2)	404 (16.8)	12 (15.4)	4 (22.2)	

*Denotes significant association at 5% level of significance.

^a^Likelihood Ratio test.

^b^Chi-square.

### Association between demographic and environmental characteristics and the severity of fall

In Table 2, examining the association of age and severity of fall, using age bracket (< 50 years) as the reference group, the results from the multivariable model indicated that individuals in the age brackets 65–74 years were 19.5% more likely to fall at a higher severity level (*AOR = 1.195, 95% CI 1.044–1.367, p* = *0.010*), 75–83 years were 29.3% more likely to fall at a higher severity level (*AOR* = *1.293, 95% CI 1.133, 1.477, p* < *0.001*) and > 84 years were 39.1% more likely to fall at a higher severity level (*AOR* = *1.391, 95% CI 1.210–1.599, p* < *0.001*). Females were 15.1% more likely to fall at higher severity level compared to males (*AOR* = *1.151, 95% CI 1.063, 1.247, p* < *0.001*). With respect to activity at time of the fall, incidents during toileting and showering activities were 14.5% more likely to fall in higher level of severity (*AOR* = *1.145, 95% CI 1.022, 1.284, p* = *0.020*) compared with attempting to sit or stand. Using bathroom as the reference point, fall incident in a communal area was approximately 25.7% more likely to fall in higher level of severity (*AOR* = *1.257, 95% CI 1.003, 1.576, p* = *0.047*). In the univariable analysis using low fall height (< 0.5 m) as the reference group, fall incidents with unknown height were 18.1% more likely to be associated with a higher level of severity (*AOR* = *1.181, 95% CI 1.015, 1.374, p* = *0.031*). However, none of the height of falls were found significant in the multivariable model.

**Table 2:** Univariable and multivariable analysis of demographic and environmental risk factors associated with the severity level of fall.

Variables	Univariable analysis			Multivariable analysis		
	Est. (SE)	Odds (95% CI)	p-value	Est. (SE)	Adj. Odds (95% CI)	p-value
**Intercept**				− 1.889 (0.078)	0.151 (0.130–0.176)	< 0.001*
**Age**						
< 50	0	1	–	0	1	–
50–64	− 0.065 (0.065)	0.937 (0.824, 1.065)	0.320	− 0.051 (0.068)	0.951 (0.832, 1.086)	0.457
65–74	0.149 (0.066)	1.161 (1.020, 1.321)	0.023*	0.178 (0.069)	1.195 (1.044, 1.367)	0.010*
75–84	0.250 (0.065)	1.284 (1.131, 1.459)	< 0.001*	0.257 (0.068)	1.293 (1.133, 1.477)	< 0.001*
> 84	0.328 (0.068)	1.389 (1.231, 1.601)	< 0.001*	0.330 (0.071)	1.391 (1.210, 1.599)	< 0.001*
**Gender**						
Male	0	1	–	0	1	–
Female	0.154 (0.039)	1.166 (1.080, 1.260)	< 0.001*	0.141 (0.041)	1.151 (1.063, 1.247)	0.001*
**Activity at fall**						
Attempting to sit/stand/bending and reaching over	0	1	–	0	1	–
Getting in/out of bed	0.040 (0.057)	1.041 (0.932, 1.163)	0.476	0.068 (0.059)	1.070 (0.953, 1.201)	0.251
Toileting and showering	0.162 (0.522)	1.175 (1.061, 1.303)	0.002*	0.136 (0.058)	1.145 (1.022, 1.284)	0.020*
Walking and running	0.015 (0.056)	1.015 (0.909, 1.134)	0.787	− 0.062 (0.061)	0.940 (0.834, 1.060)	0.311
Unknown	0.135 (0.074)	1.144 (0.990, 1.322)	0.067	0.160 (0.115)	1.174 (0.938, 1.470)	0.161
**Place of incident**						
Bathroom	0	1	–	0	1	–
Bed	− 0.148 (0.053)	0.862 (0.777, 0.956)	0.005*	− 0.112 (0.060)	0.894 (0.794, 1.006)	0.062
Allied health treatment area	− 0.040 (0.052)	0.960 (0.867, 1.064)	0.441	0.002 (0.058)	1.002 (0.895, 1.123)	0.969
Communal area	0.144 (0.111)	1.155 (0.929, 1.437)	0.195	0.229 (0.115)	1.257 (1.003, 1.576)	0.047*
**Height of fall**						
Low fall (< 0.5 m)	0	1	–	0	1	–
Medium fall (0.5–1.0 m)	0.071 (0.043)	1.074 (0.988, 1.168)	0.094	0.084 (0.045)	1.088 (0.996, 1.189)	0.063
High fall (> 1.0 m)	− 0.103 (0.220)	0.902 (0.586, 1.390)	0.641	− 0.020 (0.221)	0.980 (0.636, 1.151)	0.928
Unknown	0.166 (0.077)	1.181 (1.015, 1.374)	0.031*	0.128 (0.079)	1.136 (0.973, 1.327)	0.107

*Denotes significant association at 5% level of significance.

### Association between patient’s medical and health risk factors and the level of severity of fall

The association between severity of falls and patients’ pre-diagnosed medical and health risk factors were examined in Table 3. Patients with risk condition present were set as reference group in the model. Under behavioural risk factors, marked depression was positively associated with severity of fall incidents. Patients with depression had over 167% increase in the odds of falling at higher severity level compared to those without depression *(AOR* = *0.374, 95% CI 0.184, 0.760, p* = *0.007)*. Patients under the influence of alcohol or illicit drugs had the similar trend of falling at higher level of severity. Empirically, patients under the influence of alcohol or illicit drugs had a significantly higher (more than 300% increase) odds of falling at a higher level of severity compared to those who have no alcohol or illicit drugs in their system *(AOR* = *0.234, 95% CI 0.116, 0.472, p* ≤ *0.001)*.

**Table 3:** Multivariable analysis of patient’s medical and health risk factors associated with the severity level of fall.

Risk category	Risk condition	SAC 3 *n (%)* (n = 3545)	SAC 2 *n (%) *(n = 142)	SAC 1 *n (%)* (n = 18)	Estimate (SD)	p-value	Adj. odds [95% confidence interval]
History of fall	No falls history	1,414 (37.0)	52 (36.6)	10 (55.6)	− 0.010 (0.262)	0.969	0.990 [0.593, 1.653]
	> 1 fall in previous 6 months	1,472 (38.5)	47 (33.1)	5 (27.8)	0.192 (0.222)	0.387	1.212 [0.784, 1.873]
	Admitted because of a fall	638 (16.7)	27 (19.0)	4 (22.2)	− 0.122 (0.238)	0.608	0.885 [0.555, 1.411]
	*Had fall/s or near miss/es during current admission*	775 (20.3)	34 (23.9)	3 (16.7)	− 0.399 (0.237)	0.092	0.671 [0.422, 1.067]
Behavioural	No behaviour/mental state/cognition issues	1,049 (27.4)	40 (28.2)	6 (33.3)	− 0.113 (0.250)	0.652	0.893 [0.547, 1.459]
	Dehydration	226 (5.9)	6 (4.2)	1 (5.6)	0.352 (0.424)	0.406	1.422 [0.620, 3.260]
	Delirium, anxiety, agitation issues	1,072 (28.0)	35 (24.6)	6 (33.3)	0.001 (0.217)	0.996	1.001 [0.654, 1.531]
	Dementia/cognitive impairment issues	1,014 (26.5)	36 (25.4)	3 (16.7)	0.033 (0.222)	0.883	1.033 [0.669, 1.597]
	Difficulty communicating or following instructions	1,045 (27.3)	35 (24.6)	4 (22.2)	0.021 (0.217)	0.922	1.022 [0.667, 1.563]
	Impaired consciousness	192 (5.0)	10 (7.0)	1 (5.6)	− 0.531 (0.344)	0.123	0.588 [0.300, 1.154]
	Intellectual disability affecting judgement of physical ability	157 (4.1)	2 (1.4)	1 (5.6)	0.500 (0.602)	0.406	1.649 [0.507, 5.363]
	Marked depression	131 (3.4)	9 (6.3)	2 (11.1)	− 0.983 (0.362)	0.007*	0.374 [0.184, 0.760]
	Neurological condition	750 (19.6)	27 (19.0)	2 (11.1)	− 0.195 (0.270)	0.470	0.823 [0.484, 1.398]
	Under the influence of alcohol or illicit drugs	105 (2.7)	13 (9.2)	0 (0.0)	− 1.452 (0.357)	< 0.001*	0.234 [0.116, 0.472]
Mobility	No mobility transfer issues	217 (5.7)	13 (9.2)	0 (0.0)	0.183 (0.344)	0.594	1.201 [0.612, 2.355]
	Dizziness, light-headedness, faintness, dehydration	541 (14.2)	21 (14.8)	0 (0.0)	0.117 (0.256)	0.647	1.124 [0.681, 1.855]
	Impaired lower limb peripheral sensation	486 (12.7)	12 (8.5)	3 (16.7)	0.249 (0.303)	0.411	1.283 [0.708, 2.325]
	Lower limb amputee	76 (2.0)	1 (0.7)	0 (0.0)	1.149 (1.022)	0.261	3.155 [0.425, 23.392]
	Non or partial weight-bearing	274 (7.2)	6 (4.2)	1 (5.6)	0.587 (0.418)	0.160	1.798 [0.793, 4.077]
	Poor balance, unsteady	2,333 (61.0)	70 (49.3)	12 (66.7)	0.483 (0.192)	0.012*	1.622 [1.114, 2.361]
	Poor vision, such that it affects mobility	283 (7.4)	11 (7.7)	1 (5.6)	0.031 (0.318)	0.922	1.032 [0.553, 1.925]
	Requires assistance to mobilise	1,727 (45.2)	56 (39.4)	9 (50.0)	0.051 (0.197)	0.796	1.052 [0.715, 1.549]
	Requires standby assistance	1,500 (39.2)	39 (27.5)	11 (61.1)	0.387 (0.190)	0.042*	1.473 [1.014, 2.138]
	Requires walking aid or similar e.g., crutches, walking frame	1,462 (38.2)	49 (34.5)	10 (55.6)	− 0.073 (0.197)	0.711	0.930 [0.632, 1.368]
	Severe foot problems—pain, deformity, or marked swelling	249 (6.5)	12 (8.5)	1 (5.6)	− 0.482 (0.334)	0.149	0.618 [0.321, 1.188]
	Significant pain when walking, transferring	253 (6.6)	13 (9.2)	1 (5.6)	− 0.445 (0.312)	0.154	0.641 [0.348, 1.182]
	Urge incontinence, occasional incontinence	590 (15.4)	22 (15.5)	4 (22.2)	− 0.204 (0.235)	0.384	0.815 [0.515, 1.291]
	Weakness, generalised muscular weakness	1,142 (29.9)	31 (21.8)	7 (38.9)	0.266 (0.205)	0.195	1.305 [0.873, 1.951]
Medication	Medication issues	1,061 (27.8)	55 (38.7)	5 (27.8)	− 0.623 (0.260)	0.017*	0.537 [0.322, 0.893]
	Diuretics	490 (12.8)	17 (12.0)	2 (11.1)	− 0.071 (0.275)	0.797	0.932 [0.543, 1.598]
	General Anaesthetic (within 24/24)	69 (1.8)	5 (3.5)	0 (0.0)	− 0.754 (0.502)	0.133	0.470 [0.176, 1.257]
	Polypharmacy—more than 5 prescribed medications	1,877 (49.1)	59 (41.5)	12 (66.7)	− 0.297 (0.243)	0.221	0.743 [0.462, 1.196]
	Psychoactive medications—antidepressants or benzodiazepines	654 (17.1)	21 (14.8)	2 (11.1)	0.180 (0.268)	0.501	1.197 [0.708, 2.023]
	Sedation within 12/24 of assessment	304 (8.0)	8 (5.6)	0 (0.0)	0.423 (0.389)	0.277	1.527 [0.712, 3.274]
	Substantial change to medication regime	118 (3.1)	1 (0.7)	1 (5.6)	0.682 (0.727)	0.348	1.978 [0.476, 8.222]
Physiological	Anticoagulant therapy/bleeding disorder	1650 ()	49 ()	12 (66.7)	0.124 (0.205)	0.545	1.133 [0.757, 1.694]
	Diagnosis of osteoporosis	118 3.1)	5 (3.5)	0 (0.0)	− 0.124 (0.447)	0.782	0.884 [0.368, 2.121]
	Fragile skin	1318 (37.2)	61 (43.0)	10 (55.6)	− 0.559 (0.214)	0.001*	0.572 [0.376, 0.869]

NB: Patients with risk condition present were set as reference group in the model. *Denotes significant association at 5% level of significance. The reported adjusted odds estimates are also age-gender adjusted.

In relation to mobility, patients who did not required standby assistance were 47.3% *(AOR = 1.473, 95% CI 1.014, 2.138, p = 0.042)* more likely to experience higher severity fall compared to those who required assistance. In practice patients who have poor or unsteady balance will seek for assistance. Effectively, we found that patients with poor or unsteady balance were 38.3% less likely to fall at a higher level of severity (*AOR* = *1.622, 95% CI 1.114, 2.361, p* = *0.012*) compared to patients who did not have the condition. In relation to medication issues, there was an 86% increase in the odds in patients who were on medication to fall at high severity compared to patients who are not on medications (*AOR* = *0.537, 95% CI 0.322, 0.893, p* = *0.017*). For instance, polypharmacy, (although not found to be statistically significant) contributed to higher odds (35% increase in likelihood) (*AOR* = *0.743, 95% CI 0.462, 1.196, p* = *0.221*) of falling at a high severity level. There was approximately 75% increase in the odds of patients with fragile skin condition to fall at a higher severity level compared to patients without the condition (*AOR* = *0.572, 95% CI 0.376, 0.869, p* = *0.001*).

## Discussion

This study examined the factors that are associated with the severity of patient falls, using five years of data extracted from a clinical incident database in an acute hospital setting. Therefore, it was practical to examine the associated factors which may provide an opportunity to improve policy and procedure and develop intervention by identifying the severity of falls which can cause serious injuries and harm. In this study, the risk of falling with a higher level of injury-severity increased by approximately 20% for patients aged 65–74 years, 29% for patients aged 75–83 years and 39% for patients aged over 84 when compared to patients who were aged 50 years or younger. The results of increasing rate of falling with higher severity with increasing age is consistent with other studies which indicated that older the patient, the higher the odds of a fall^6,28,29^. Elderly patients might also be vulnerable to high level of fall injuries due to co-existing health problems compared with their younger counterparts. In this study, gender was a significant risk factor, where females were 15.1% more likely to fall in higher severity condition. While gender-specific risk factor is not common in inpatient-falls research, the proportion of injurious falls reported to be much higher among females than males in aging population in community dwelling-houses^30,31^.

Fall from a height was 1.2 times more common in elderly adult female patients leading to traumatic brain injuries requiring hospital admission^32^. Factors such as stroke, age of 85 years or older, nutritional risk, consumption of alcohol, use polypharmacy, arthritis, diabetes and osteoporosis were found to be independently correlated with female fallers^33^. The cause for gender differences in fall-related injury is unclear. Gender differences in biomechanical differences in the gait pattern^34^ could be a critical factor which was reported associated with knee osteoarthritis in elderly females^35^. Another possible reason could be related to footwear which can have detrimental effects on gait pattern, postural balance and other part of musculoskeletal system which cause females for falls and fall-injuries than males^36,37^. Females have a longer life expectancy than males and so are more likely to have an increased need for nursing or residential home care^38,39^. This also could explain the higher falls among females in aged care residents.

In our study, it was observed that falls occurring from toileting and showering activities were 14.5% more likely to result in a higher level of fall severity compared to attempting to sit, stand or reaching over. Patient falls specifically related to bathroom activities is of particular concern. Bathroom activities have resulted in 38–47% of falls in US hospitals^40^. Many of the falls with injuries were directly related to toileting or showering^41^. In another study, fall injuries were 2.48 times greater if a fall occurred in bathroom^4^. Fall incident in communal areas was approximately 26% more likely to fall in higher level of severity. Falls were found more likely than expected to be occurred in communal areas in a previous study^42^. These communal areas are more likely to be unattended and un-witnessed by nurses, and/or lack in risk assessment and falls prevention interventions in place. Kobayashi et al.^43^ reported that falls occurred in waiting room due to the arrangement of chairs, and slippery mats at entrances, and in parking places due to its distance from hospital wards and stairs. Falls were also occurred in the passage and dining room where no call bell was available^44^.

Under behavioural factors, depression and alcohol or illicit drug were major risk factors for fall incidents and significantly contributed to the level of severity. Similar to our study, patients with depression were reported to have increased odds for in-hospital fall related major injuries^45^. Depression and the use of antidepressant drug was found to increase falls risk^46,47^. Illicit drug use was reported to be associated with increased odds of injurious falls in patients living with HIV^48^. However, the association between history of consumption of alcohol and falling was not evident in community-dwelling older adults^49–51^. The relationship of alcohol consumption and falls in community and hospital settings deserves future investigations.

Mobility impairment due to poor and unsteady balance and patients who required standby assistance revealed to be significant risk factors for the severity of falls. The results were consistent with other studies which showed mobility impairment and activities with daily living dependency as higher risk of falls^28,29^. The results suggest that patients with poor and unsteady balance need standby assistance for constant monitoring and guiding their movement to avoid serious injurious falls. Patients with medication issues were more likely to fall at higher severity. Evidently, patients with concurrent use of five or more medicines in this study had an increased odds of falling at high severity. Polypharmacy was reported to be significantly associated with an increased risk of falls and fractures in elderly^52–54^. The significant association of polypharmacy for severity of falls was further evident in nursing home residents^55,56^ and community settings^49^. Patients’ routine medication reviews need to be considered to mitigate the severity of fall and fall-related injury during their hospital stay.

The significant association between fragile skin and higher fall severity in this study is unclear. One explanation is that fragile skin or thin skin is a common problem due to aging which leads to serious implications for health and wellbeing of the elderly including skin tear^57,58^. Fragile skin thus increased the risk of injuries once a fall occurred in the hospital. The aged population thus needs to be closely monitored to avoid high fall severity and serious fall injury. The association between fragility of the skin and fall severity is new findings and requires further investigation.

## Limitations

As the clinical incident reports are completed by a large number of nurses with various level of experience and different backgrounds, some classification of patient information can be prone to a variation in clinical judgement. As the study was conducted in one teaching hospital, generalisability of the finding is limited to similar acute care hospital settings. However, one can anticipate that severity of falls and associated risk factors would be similar at other major metropolitan teaching hospitals. Future studies should also consider matching faller and non-faller groups on selected demographic variables to compare similarities and differences on the risk factors found to be significant. Future studies may consider examining disease-specific risk factors for severity of falls.

### Implication for clinical practices

Preventing patient falls is an important endeavour and continues to be of interest to nurse leaders, clinicians, and researchers. Health care facilities have introduced robust falls prevention strategies such as screening patients to establish their level of risk and fall prevention care plans to reduce the number of patient falls; however, patients continue to fall. It may be that preventing falls is a difficult task unless there is the capacity for twenty-four-hour supervision, which is unrealistic. Consequently, the focus should be on how nurses and clinicians reduce severity of falls in hospitals. The results have highlighted significant risk factors that must be considered carefully in fall management of populations like cohort in our study. Additionally, the adjusted odds reported for all the risk conditions will inform practice, even for the non-significant risk conditions, as clinical significance and statistical significance may vary sometimes. The findings may guide nurse leaders and clinicians to develop successful hospital-based interventions and strategies to prevent severity of falls and fall-related serious injury in acute hospitals. The findings of this study further add to the fall literature identifying the major factors associated with increased severity of falls. It may be useful to provide both patients and the general community with written and media information of these issues so they can independently be more vigilant about their own risk factors and how to be more proactive about falls prevention. This may be a more efficient strategy worthy of further exploration.

## Conclusion

This study provides information on factors associated with the severity of falls over a 5-year period in an acute care hospital. The results showed multivariable factors of increased age, being female, toileting and showering were all associated with increased severity of a fall. Additionally, depression under the influence of alcohol or drugs, poor balance and requiring standby assistance were also associated with increased severity of falls. The findings suggest that assessments of severity of fall risk should weigh these variables which are associated with the severity of fall identified in this study. Interventions are recommended to be developed or implemented based on these variables. Given the differences in the severity of falls by age and gender, hospital executive must consider that a one size fits all approach is not effective when developing and implementing severity of falls-prevention strategies at both intrinsic and extrinsic level. Identification of underlying risk factors associated with the severity of falls may provide information that can inform the implementation of fall prevention strategies that mitigate the risk of injurious falls.

## Data Availability

WA Health and Edith Cowan University Human Research Ethics Committee does not permit the authors to make data publicly available.

## Abbreviations

AOR: Adjusted Odds Ratio
CI: Confidence Interval
CIM: Clinical Incident Management
DOHWA: Department of Health Western Australia
ECU: Edith Cowan University
SCGOPHCG: Sir Charles Gairdner Osborne Park Health Care Group
WA: Western Australia
